# Unraveling the Metabolic and Molecular Basis of Floral Pigmentation Shift in *Nymphaea atrans*

**DOI:** 10.3390/genes17040442

**Published:** 2026-04-12

**Authors:** Qian Wei, Kaijie Zhou, Mengchao Fang, Zhentao Ren, Shujuan Li, Ming Zhu

**Affiliations:** 1 Xi’an Botanical Garden of Shaanxi Province, Institute of Botany of Shaanxi Province, Shaanxi Engineering Research Centre for Conservation and Utilization of Botanical Resources, Xi’an 710061, China; weiqian@xab.ac.cn (Q.W.); lishujuanxbg@163.com (S.L.); 2 College Biological Science and Food Engineering, Southwest Forestry University, Kunming 650224, China; imkennethzhou@126.com; 3 School of Geography and Planning, Chizhou University, Chizhou 247000, China; fangmch@outlook.com; 4 Institute of Food and Nutrition Development, Ministry of Agriculture and Rural Affairs, Beijing 100081, China; rztkkk@163.com; 5 College of Open Education, Yunnan Open University, Kunming 650032, China

**Keywords:** *Nymphaea atrans*, flower color, anthocyanin, jasmonic acid signaling

## Abstract

**Background**: *Nymphaea atrans* exhibits a gradual flower color transition from nearly white to rose-red during anthesis, yet the molecular mechanisms of this phenomenon remain unclear. In the present study, transcriptomic and metabolomic analyses were performed to systematically investigate anthocyanin accumulation patterns and regulatory mechanisms during the color transition of *N. atrans*. **Methods**: Petals were collected at three flowering stages: day 1 (D1), day 3 (D3), and day 5 (D5). Targeted metabolomics was performed using UPLC-ESI-MS/MS to profile anthocyanin and other flavonoid metabolites. Transcriptome analysis was conducted via RNA-seq. Differentially accumulated metabolites (DAMs) and differentially expressed genes (DEGs) were identified, followed by functional enrichment and integration analysis. **Results**: The results revealed significant accumulation of seven anthocyanins, including cyanidin-3-O-arabinoside, cyanidin-3-O-glucoside, cyanidin-3-O-galactoside, cyanidin-3-O-(6″-O-acetyl)-glucoside, at stages D3 (day 3 after flowering, light pink petals) and D5 (day 5 after flowering, deep pink petals), accompanied by the upregulation of key enzyme-encoding genes, chalcone synthase, chalcone isomerase, flavanone 3-hydroxylase, di-hydroflavonol 4-reductase, and anthocyanidin synthase in the anthocyanin biosynthetic pathway. Genes involved in JA biosynthesis and key regulatory genes in the JA signaling pathway were significantly up-regulated, indicating that the JA signaling pathway may play an important regulatory role in the synthesis of anthocyanins in *N. atrans*. **Conclusions**: This study unravels the metabolic and molecular underpinnings of flower color transition in *N. atrans*, thereby establishing a theoretical basis for the targeted regulation of floral pigmentation and molecular breeding of ornamental water lilies.

## 1. Introduction

In ornamental horticultural plants, flower color largely determines aesthetic appreciation and practical use, ranking among their most distinctive traits [1,2]. The formation of flower color is determined not only by genetic factors but also by developmental stages, environmental conditions, and hormonal signaling pathways [1,2,3,4]. Water lilies (*Nymphaea* spp.), as globally important aquatic ornamentals, have attracted considerable attention due to their diverse flower colors and unique blooming characteristics [5,6]. Water lilies are globally important aquatic ornamentals with high horticultural and cultural value. *N. atrans*, a species native to tropical Asia, belongs to the subgenus Brachyceras and is characterized by its unique flower color transition from white to rose-red during anthesis. Within the Nymphaeaceae family, such dynamic color changes are also observed in Victoria species, making *N. atrans* an excellent model to study the molecular basis of floral pigmentation shifts. In China, the water lily market has grown steadily, reaching an estimated RMB 5.2 billion by 2024. The annual production of water lilies in China has been continuously increasing. According to data from the China Flower Association, by the end of 2019, the annual production of water lilies in China had reached 152 million plants, with the total market size of the lotus and water lily industry reaching RMB 10 billion. *Nymphaea atrans* is a unique water lily, distinguished by its flower color gradually transitioning from nearly white to rose-red during anthesis. A similar flower color change has also been observed in its closely related species, *Victoria cruziana*. This distinctive temporal variation in petal color positions *N. atrans* as an optimal model for elucidating the genetic and biochemical pathways underlying floral color formation. Although no reference genome has been published for *N. atrans* to date, a high-quality genome is available for the closely related species *Nymphaea colorata* [5], and transcriptomic datasets have been reported for multiple water lily species [6]. However, the metabolic and regulatory networks of color transition remain unknown in *N. atrans*.

Floral coloration in the majority of angiosperms is primarily attributable to anthocyanin pigments [7,8]. Variations in anthocyanin composition and accumulation levels directly affect the visual presentation of petal colors [9]. The enzymatic cascade responsible for anthocyanin biosynthesis has been well characterized across multiple plant lineages, featuring key catalysts such as chalcone synthase (*CHS*), flavanone 3-hydroxylase (*F3H*), and flavonoid 3-O-glucosyltransferase (*UFGT*) [10,11,12]. At the regulatory level, anthocyanin biosynthesis is tightly controlled by a network of transcription factors, among which the MYB-bHLH-WD40 (MBW) complex plays a central role [13,14,15]. In Actinidia chinensis, the MBW complex (AcMYBF110–AcbHLH1–AcWDR1) regulated anthocyanin biosynthesis by activating genes, such as *AcCHS*, *AcF3′H*, *AcANS*, *AcUFGT3a*, and *AcUFGT6b* [16]. MYB transcription factors are pivotal regulators that direct anthocyanin biosynthesis across diverse plant lineages. The *AtMYB75* gene belongs to the S6 subgroup of R2R3-MYB gene family in *Arabidopsis*, which is known to regulate anthocyanin biosynthesis [17,18,19]. In recent years, plant hormones have emerged as critical regulators of flower color formation, with the jasmonic acid (JA) signaling pathway receiving particular attention [20,21]. JA has been reported to regulate anthocyanin accumulation by regulating flavonoid-related transcription factors through core components of the JA signaling pathway, such as COI1, JAZ, and MYC2, which in turn orchestrate the expression of structural loci [22,23]. Although such mechanisms have been described in plants like *Arabidopsis* [17], grapevine [24], and apple [25], the involvement of JA in anthocyanin biosynthesis regulation in water lilies has not been systematically studied.

This study recorded the progressive pigmentation change in *N. atrans* and leveraged multi-omics (metabolomic and transcriptomic) analyses of petal tissues at different flowering time points to dissect both anthocyanin buildup and the corresponding transcriptional dynamics of core biosynthetic genes. Moreover, the role of JA biosynthesis and signaling pathways in regulating anthocyanin production was analyzed in *N. atrans*, offering novel perspectives on the molecular basis of floral pigmentation shifts in water lilies.

## 2. Results

### 2.1. Dynamic Changes in Floral Color Phenotypes and CIELAB Color Space Analysis

**Figure 1:**
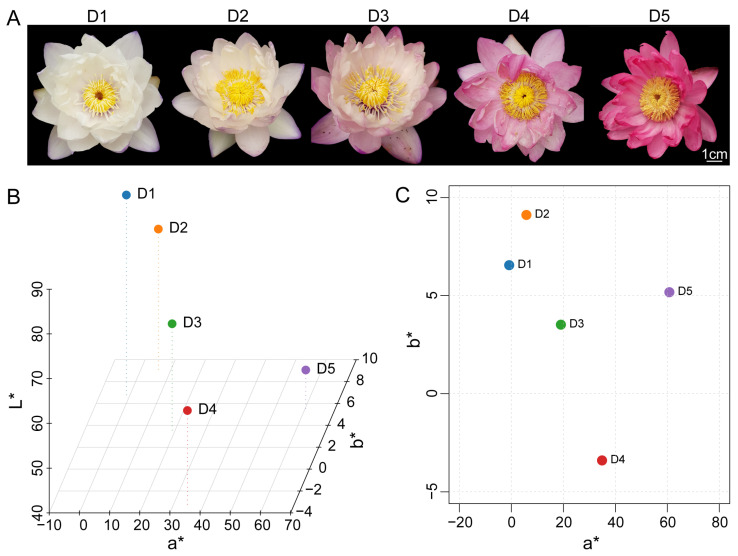
Dynamic changes in petal color of *N. atrans* and CIELAB color space analysis. (**A**) Color changes in flowers from D1 to D5, showing a gradual transition from near-white to red. (**B**) 3-dimensional (3D) scatter plot of L*, a*, and b* values at different stages of flowering. (**C**) 2-dimensional (2D) scatter plot of a* and b* values illustrating the shift in petal color towards the red spectrum over time. D1, D2, D3, D4, and D5 represent day 1, day 2, day 3, day 4, and day 5 after flowering, respectively.

To systematically analyze the dynamic changes in petal color during the flowering of *N. atrans*, quantitative characterization was performed using the CIELAB color space. From day 1 (D1) to day 5 (D5), the petal color transitioned from near-white to magenta (Figure 1A). The L* values continuously decreased from D1 to D5, indicating that petal brightness declined and the color gradually deepened over time (Figure 1B). The a* values significantly increased from −0.844 on day 1 to 60.772 on day 5, reflecting a continuous accumulation of red pigments (Figure 1B,C). In contrast, b* values exhibited only minor fluctuations during flowering (Figure 1B,C). The CIELAB analysis demonstrated that the color transition in *N. atrans* petals is primarily driven by the accumulation of red pigments, consistent with the observed visual changes from white to pink and ultimately to red. It should be noted that the staging (D1, D3, D5) was defined based on the flower color transition observed under the specific growth conditions employed in this study (Haikou, outdoor pond, natural light and temperature). The exact timing may vary depending on environmental factors such as light intensity and temperature; nevertheless, the sequential pattern of color transition remains consistent.

### 2.2. Metabolomic Profiling of N. atrans Petals

**Figure 2:**
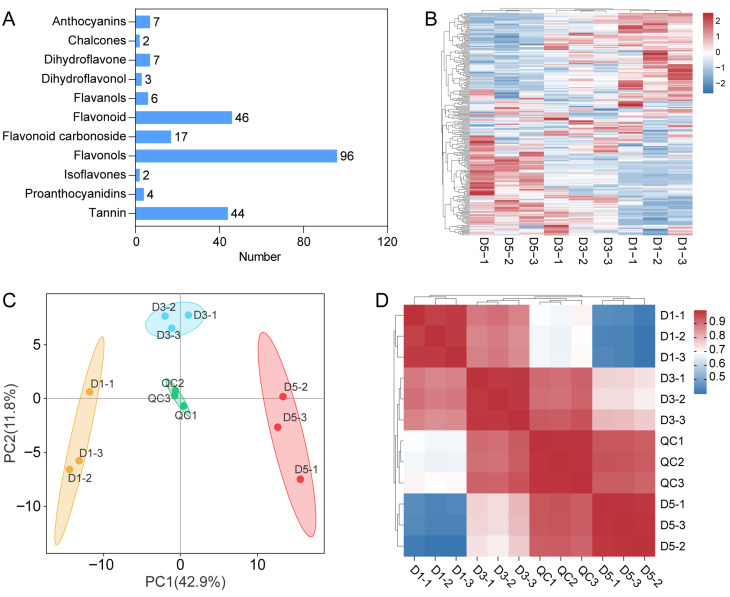
Metabolite composition and global differences in *N. atrans* petals. (**A**) Classification of detected metabolites. (**B**) Heatmap of metabolite profiles across developmental stages. (**C**) PCA-based ordination of metabolomic profiles. (**D**) Hierarchical clustering analysis of petal metabolite profiles. The heatmap shows relative metabolite abundance (scaled by row). The left-side dendrogram clusters metabolites into major classes including flavonols (e.g., quercetin derivatives), flavonoids (e.g., luteolin derivatives), anthocyanins, phenolic acids, and others. Red indicates high abundance, blue indicates low abundance. Three biological replicates per stage are shown.

To further elucidate the biochemical basis underlying the petal color changes in *N. atrans*, targeted metabolomic analyses were conducted on petal tissues collected at D1, day3 (D3), and D5 stages, based on phenotypic and CIELAB data. The analysis yielded 234 metabolites, which were sorted into 11 categories by structural and functional criteria. Flavonols accounted for the largest proportion, followed by flavonoids, highlighting the significant accumulation of flavonoid compounds during petal development (Figure 2A). The metabolite heatmap revealed marked differences in metabolite accumulation among samples at different stages (Figure 2B). Principal component analysis (PCA) showed clear separation of samples along PC1, indicating distinct metabolic profiles between stages (Figure 2C). Hierarchical clustering analysis further confirmed substantial differences in metabolite accumulation patterns, particularly between D1 and D5 samples (Figure 2D).

### 2.3. Identification of Differential Metabolites Reveals Anthocyanins as Key Drivers of Color Change

**Figure 3:**
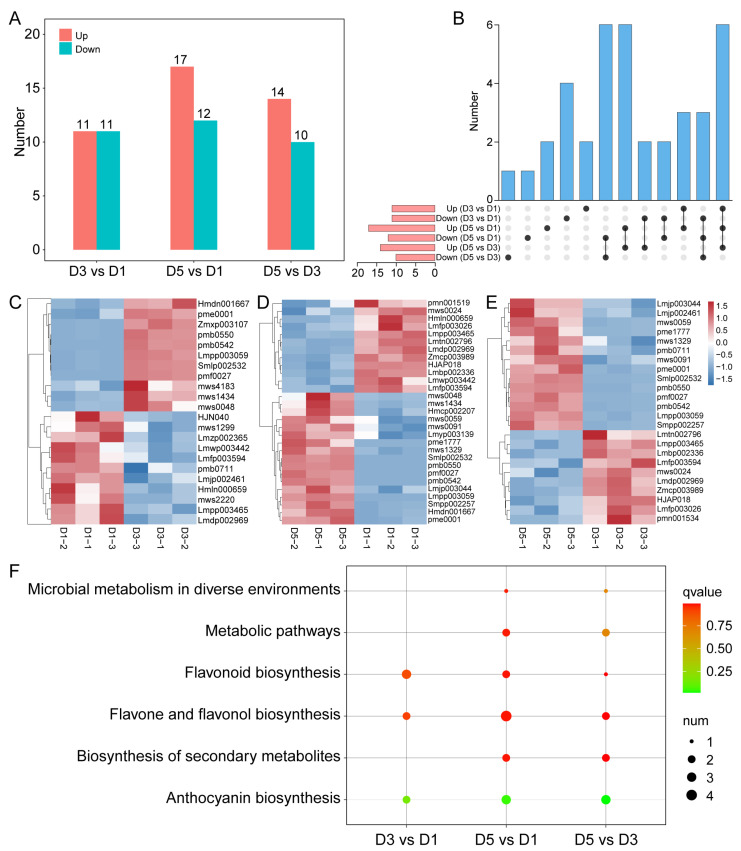
Differential metabolite analysis of *N. atrans* petals. (**A**) Statistics of DAMs in pairwise comparisons. (**B**) Updated to indicate both up and down DAMs. (**C**) Heatmap of DAM expression in D3 vs. D1. (**D**) Heatmap of DAM expression in D5 vs. D1. (**E**) Heatmap of DAM expression in D5 vs. D3. (**F**) KEGG pathway enrichment analysis of DAMs.

Differentially accumulated metabolites (DAMs) were identified to pinpoint the key metabolites involved in the *N. atrans* petal color transition. PCA confirmed significant differences in metabolic profiles between developmental stages with high intra-group reproducibility, and permutation testing verified the model’s consistency and reliability, showing no evidence of overfitting (Figure S1). A total of 11, 17, and 14 upregulated DAMs were identified in D3 vs. D1, D5 vs. D1, and D5 vs. D3 comparisons, respectively. Notably, six DAMs showed continuous upregulation across all three comparisons, including five representative anthocyanins, namely kuromanin, cyanidin-3-O-galactoside, and cyanidin-3-O-(6″-O-malonyl)-glucoside (Figure 3A,B), suggesting their pivotal role in the petal color transition from white to red. In addition to the six continuously upregulated DAMs, downregulated differential metabolites were also observed: specifically, 4, 7, and 5 DAMs were significantly downregulated in the comparisons of D3 vs. D1, D5 vs. D1, and D5 vs. D3, respectively. These included several flavones and flavonols, such as tricetin and isorhamnetin derivatives, suggesting a potential metabolic shift from flavonol to anthocyanin biosynthesis during the flower color transition. Further metabolomic analyses revealed that the majority of upregulated DAMs were anthocyanins, consistent with the progressive deepening of petal color observed phenotypically (Figure 3C–E). KEGG pathway enrichment analysis revealed that these upregulated DAMs were preferentially overrepresented in pathways including anthocyanin biosynthesis, flavonoid biosynthesis, and flavone/flavonol biosynthesis (Figure 3F), further supporting the dominant role of anthocyanins in petal color change.

### 2.4. Transcriptomic Profiling of N. atrans Petals

**Figure 4:**
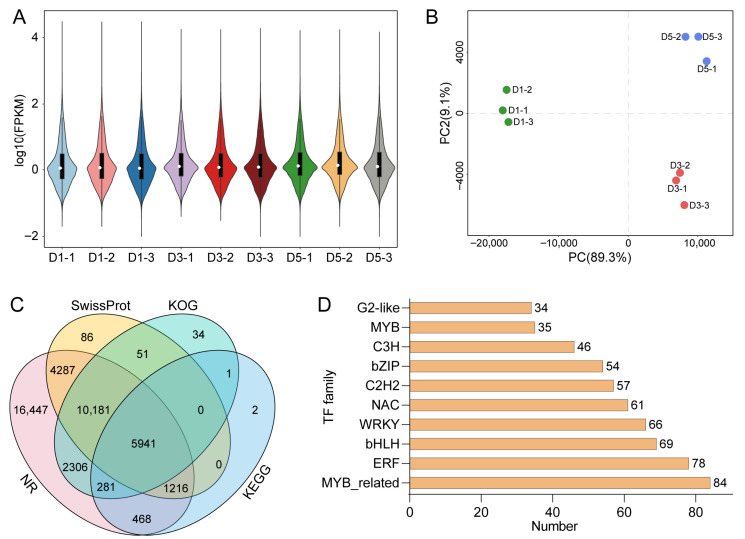
Quality assessment and functional annotation of *N. atrans* petal transcriptome. (**A**) Violin plots of gene expression levels across samples. (**B**) PCA-based dimensionality reduction of transcriptomic profiles. (**C**) Functional annotation of Unigenes. (**D**) Statistics of identified transcription factors.

To investigate the transcriptional regulatory mechanisms underlying petal color formation in *N. atrans*, transcriptome analyses were performed on petal tissues at D1, D3, and D5 stages. Violin plots showed even distribution of gene expression levels and high reproducibility within groups, demonstrating good comparability and high-quality sequencing data (Figure 4A). PCA further revealed significant differences in transcriptional profiles among developmental stages, with clear group separation (Figure 4B). Functional annotation successfully matched 54.92% of the assembled unigenes to the NR, Swiss-Prot, and KEGG databases., highlighting their potential biological relevance (Figure 4C). Additionally, 1024 transcription factors were identified from the transcriptome data, facilitating further interrogation of transcription factors that orchestrate petal color shifts (Figure 4D).

### 2.5. Differential Expression Analysis Reveals Transcriptional Dynamics Associated with Petal Color Formation

**Figure 5:**
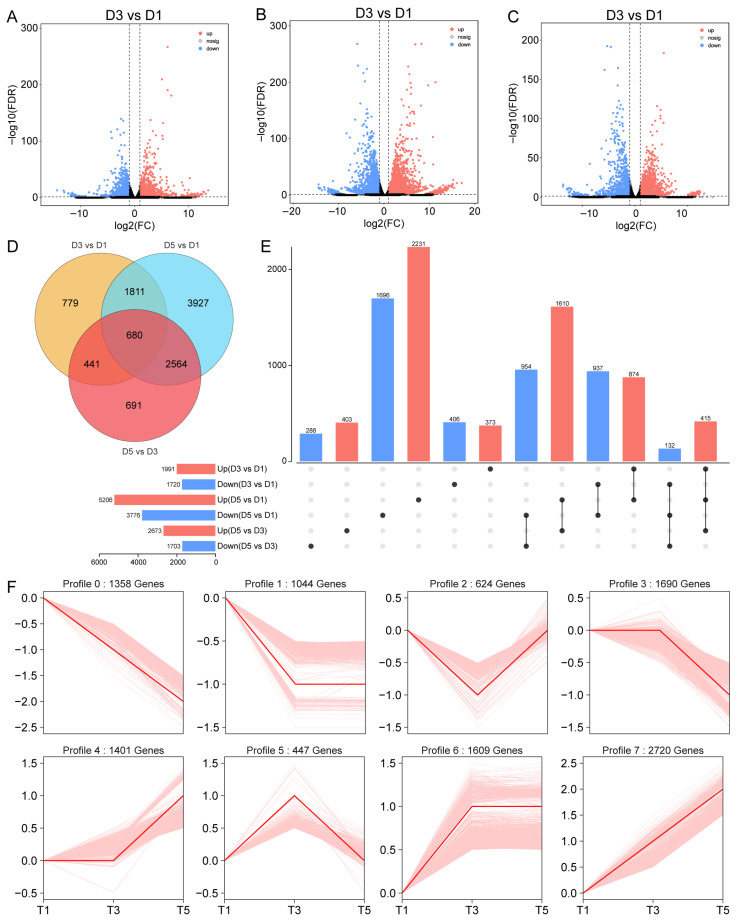
Differential expression analysis of *N. atrans* petal transcriptome. (**A**–**C**) Differential expression analyses of D3 vs. D1, D5 vs. D1, and D5 vs. D3 comparisons, respectively. (**D**) Venn plot of DEGs across comparisons. (**E**) Upset plot of DEGs across comparisons. (**F**) STEM clustering analysis of DEG expression trends.

Differential expression analysis revealed significant transcriptional changes during the petal color transition in *N. atrans*. A total of 1991, 5206, and 2673 upregulated differentially expressed genes (DEGs) were identified in D3 vs. D1, D5 vs. D1, and D5 vs. D3 comparisons, respectively (Figure 5A–C). Among these, 680 DEGs were shared across all three comparisons, including 415 genes that were continuously upregulated, suggesting their involvement in the transition from white to red petal coloration (Figure 5D,E). Among the identified DEGs, 1045, 2873, and 1218 genes were found to be downregulated in the D3 vs. D1, D5 vs. D1, and D5 vs. D3 comparisons, respectively. Notably, several of these downregulated genes were enriched in the flavonol biosynthesis pathway (e.g., FLS), which aligns with the observed reduction in specific flavonols during petal reddening. Through expression trend analysis, all DEGs were classified into eight profiles, and profiles 4, 6, and 7 exhibited upregulated expression patterns during flowering, indicating potential roles in pigment accumulation and petal color regulation (Figure 5F).

**Figure 6:**
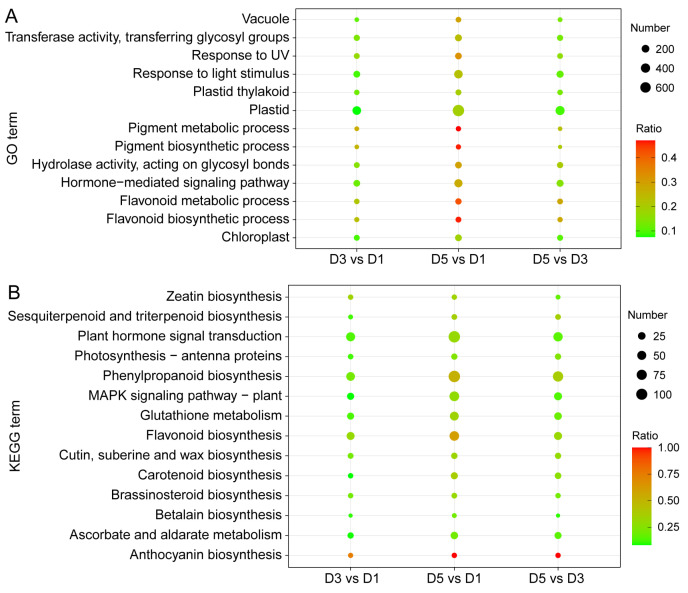
Functional enrichment analysis of *N. atrans* DEGs. (**A**) GO enrichment analysis of DEGs. (**B**) KEGG enrichment analysis of DEGs.

To further explore the functional characteristics and metabolic pathways associated with DEGs, GO and KEGG enrichment analyses were performed. GO analysis showed significant enrichment of “pigment metabolic process” and “pigment biosynthetic process”, indicating that petal color changes coincide with active pigment biosynthesis. Enrichment of “response to UV” and “response to light stimulus” suggested potential regulatory roles of environmental light in pigment accumulation. In terms of cellular components, DEGs were enriched in chloroplast, plastid, and vacuole categories, consistent with the known sites of pigment synthesis, transport, and storage, particularly the vacuole as the primary anthocyanin reservoir (Figure 6A). KEGG pathway analysis revealed that the DEGs mapped mainly to three biosynthesis pathways: flavonoid, anthocyanin, and phenylpropanoid. Notably, plant hormone signal transduction pathways were also significantly enriched, suggesting that endogenous hormones may regulate petal coloration by regulating the expression of anthocyanin-related genes (Figure 6B).

### 2.6. RT-qPCR Validation Verifies the Transcriptomic Profiles

**Figure 7:**
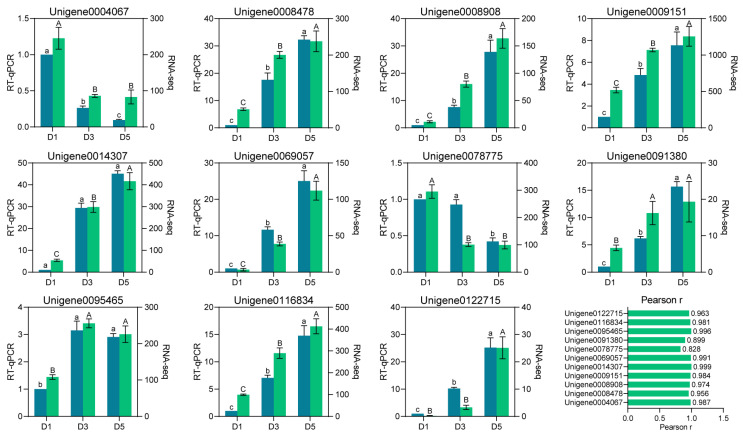
RT-qPCR validation of the expression trends of differentially expressed genes identified from the transcriptome. Gene expression levels were normalized relative to D1 (set as 1). Bar heights denote mean expression levels, with error bars representing standard deviations (n = 3). Pearson correlation coefficients (*r*) were calculated using RT-qPCR and RNA-seq expression values for each gene across the three developmental stages. Statistical significance was assessed using one-way ANOVA followed by Tukey’s post hoc test (IBM SPSS Statistics Version 19). Different lowercase letters (e.g., A, B, C, a, b, c) above the bars indicate significant differences between developmental stages for a given gene (*p* < 0.05).

The reliability of the RNA-seq findings was evaluated by randomly selecting 11 DEGs for quantitative RT-qPCR analysis at three developmental time points (D1, D3, D5), followed by a comparison of their expression trajectories with those derived from transcriptomic data. As illustrated in Figure 7, the RT-qPCR results confirmed that all tested genes displayed expression patterns at D3 and D5 that aligned well with the RNA-seq measurements. For instance, *Unigene0009151*, *Unigene0014307*, and *Unigene0116834* were upregulated at D3 and D5, whereas *Unigene0078775* and *Unigene0004067* showed a continuous downregulation during these stages. In addition, Pearson correlation analysis between the two platforms revealed that the majority of genes exhibited (*r*) values exceeding 0.95, indicating strong agreement across datasets (Figure 7). Collectively, these observations attest to the robustness and validity of the transcriptome analysis, as reinforced by RT-qPCR confirmation.

### 2.7. Integrated Transcriptomic and Metabolomic Analysis Reveals Activation of the Anthocyanin Biosynthesis Pathway

**Figure 8:**
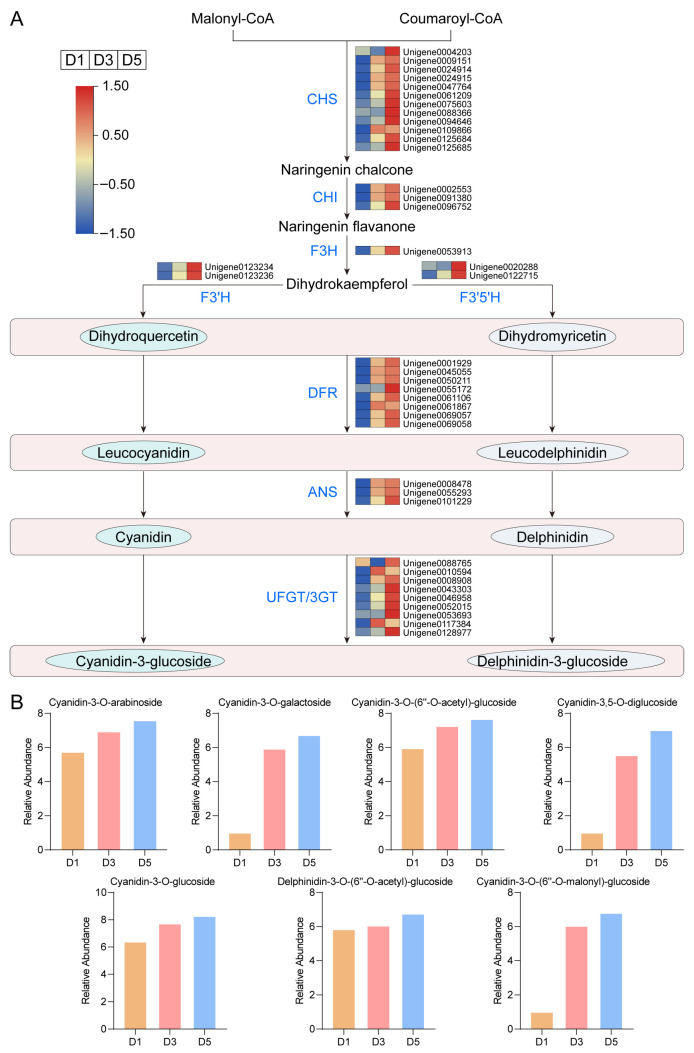
Temporal dynamics of key enzyme gene expression and concomitant anthocyanin metabolite accumulation within the anthocyanin biosynthetic pathway. (**A**) Schematic diagram of the anthocyanin biosynthetic pathway. (**B**) Accumulation dynamics of seven representative anthocyanin metabolites.

To further elucidate the molecular mechanism underlying anthocyanin accumulation during flower color transitions in *N. atrans*, transcriptomic and metabolomic data were integrated to construct a schematic diagram of the key metabolic pathway involved in anthocyanin biosynthesis (Figure 8A). In the present study, the genes encoding key enzymes in the anthocyanin biosynthesis pathway, CHS, CHI, F3H, DFR, ANS, and UFGT/3GT, were all upregulated at D3 and D5 compared to D1, indicating activation of the anthocyanin biosynthetic pathway during flower color development. Metabolomic analysis further revealed that seven anthocyanin compounds, cyanidin-3-O-arabinoside, cyanidin-3-O-glucoside, cyanidin-3-O-galactoside, cyanidin-3-O-(6″-O-acetyl)-glucoside, delphinidin-3-O-(6″-O-acetyl)-glucoside, cyanidin-3-O-(6″-O-malonyl)-glucoside, and cyanidin-3,5-O-diglucoside, accumulated to significantly higher levels in D3 and D5 petals than in D1 (Figure 8B). These metabolites changed in tandem with the expression of key anthocyanin pathway enzyme genes. Together, these integrated findings point to activation of the anthocyanin pathway—via upregulation of key biosynthetic genes and substantial anthocyanin accretion—as the principal mechanism underlying the white-to-red color shift in *N. atrans*.

### 2.8. Integrated Multi-Omics Analysis Implicates JA Signaling in Anthocyanin Regulation

**Figure 9:**
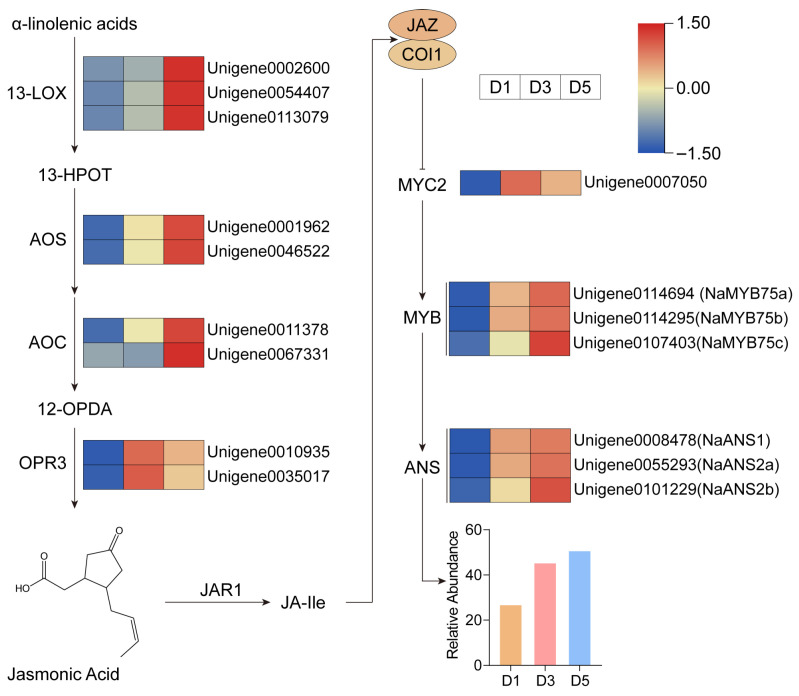
A biosynthesis pathway and JA signaling pathway in *N. atrans*.

To assess the contribution of plant hormones to flower color formation in *N. atrans*, we analyzed expression changes in JA-related genes. Transcriptome data revealed that the JA biosynthesis pathway was activated during the deepening of flower color. Specifically, the key biosynthetic enzymes, including 13-lipoxygenase (13-LOX), allene oxide synthase (AOS), allene oxide cyclase (AOC), and 12-oxophytodienoate reductase (OPR3), were all upregulated at D3 and D5, suggesting enhanced JA production during color transition (Figure 9). In the JA signaling pathway, the transcription factor *MYC2* was markedly upregulated, indicating that the JA signaling pathway was activated. In Section 2.6, RT-qPCR validation was conducted, and the results strongly support the accuracy and robustness of the transcriptome analysis. Nevertheless, these data merely enhance the observed correlation without demonstrating causality.

### 2.9. Identification of MYB Transcription Factors Regulating Anthocyanin Synthesis

**Figure 10:**
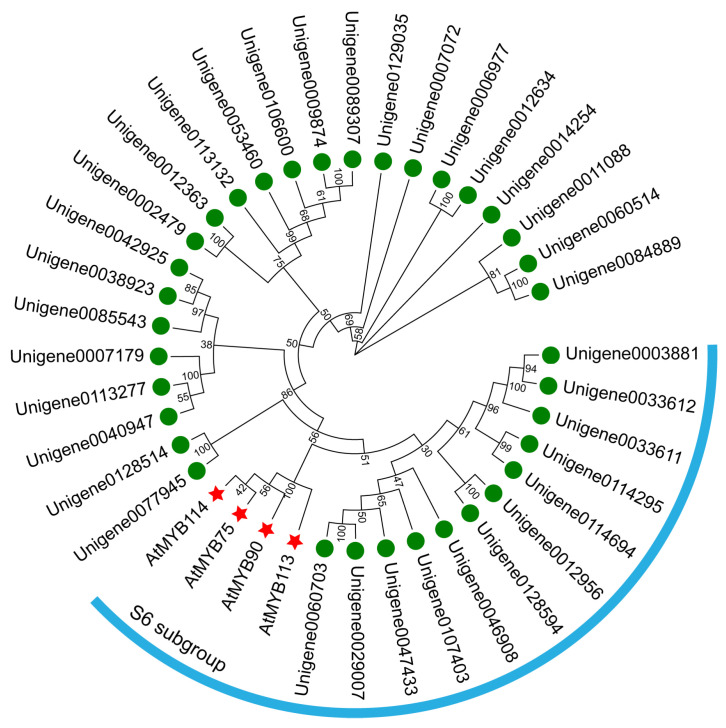
Phylogenetic analysis of S6 subgroup MYB proteins from *Arabidopsis* and MYB proteins from *N. atrans*.

In the present study, 35 MYB transcription factors were annotated in all transcripts, and we analyzed the phylogenetic relationship between these transcription factors and Arabidopsis S6 subgroup proteins. The results revealed that 12 MYB Unigenes from *N. atrans* may belong to the S6 subgroup (Figure 10), given that AtMYB75 is a regulator of anthocyanin biosynthesis, and three homologous proteins of AtMYB75 were further identified in *N. atrans*, namely Unigene0114694, Unigene0114295, and Unigene0107403, which were respectively named *NaMYB75a*, *NaMYB75b*, and *NaMYB75c*. We speculate that these three MYB transcription factors may play an important role in the flower color formation in *N. atrans*.

## 3. Discussion

The incremental saturation of flower color over the course of blooming constitutes a frequently documented event in floral organ development. In the present study, we observed a clear dynamic color change in *N. atrans*, with the flower color transitioning from nearly white at stage D1 to rose-red at stage D5. Comparable floral color shifts have been documented across diverse plant taxa. For example, in morning glory, flowers shifted from reddish-purple to blue during blooming, primarily due to a pH shift in petal vacuoles from acidic to alkaline and anthocyanin modifications [26,27]. *Hibiscus mutabilis* displays three sequential color phases—white, pink, and red—all within a single diurnal cycle [28,29]. *Victoria amazonica*, a representative species of Nymphaeaceae, is renowned for its massive leaves and unique blooming process, where the petals are white during the first night of blooming but turn pink or crimson on the second day, a change largely attributed to rapid anthocyanin accumulation [30,31]. Similarly, several tropical water lily cultivars, such as ‘Detective Erika’ [32] and ‘Feitian2’ [33], also show progressive flower color deepening during blooming. These color transitions in water lilies result from the sequential accumulation of anthocyanins or endogenous hormone-regulated mechanisms, the evolutionary significance and molecular underpinnings of which require further investigation.

In the present study, we found that the progressive reddening of *N. atrans* flowers coincided with a steady increase in anthocyanin content in the petals. Multiple anthocyanins detected in *N. atrans*, including cyanidin-3-O-arabinoside, -glucoside, -galactoside, -malonyl-glucoside, and -diglucoside, are known to confer red pigmentation in plants, and their concentrations peaked at stage D5. Anthocyanin accumulation as a driver of flower color change has also been documented in other species, such as *Lantana camara* [34], *Rhododendron sanguineum* [35], and *Chrysanthemum morifolium* [36]. Transcriptomic profiling further demonstrated that several core structural genes within the anthocyanin biosynthetic route were upregulated at D3 and D5, suggesting that activation of this pathway underlies the enhanced anthocyanin accumulation and deeper coloration observed in the petals. These findings indicated that flower color transition in *N. atrans* is a process driven by the synthesis and accumulation of anthocyanins mediated by functional genes. Similar patterns have been reported in *Paeonia lactiflora* and *Allium cepa*, where elevated expression of key anthocyanin biosynthetic genes is positively correlated with anthocyanin accumulation [37,38]. Our study extends this paradigm to the genus *Nymphaea*, confirming its applicability in water lilies.

Anthocyanin accumulation is the material basis of flower color formation and change, and its biosynthesis is influenced by multiple internal and external factors. First, transcriptional activation of key biosynthetic genes (*CHS*, *F3H*, *DFR*, and *ANS*) within the anthocyanin cascade underpins sustained pigment production. Second, the expression of these structural genes is often regulated by transcription factors, such as MYB transcription factor. In *Actinidia chinensis*, AcMYB75 transcription factor enhanced this pathway by directly binding to and activating the *AcANS* promoter [39]. Previous studies have confirmed that AtMYB75 transcription factor was an endogenous transcriptional regulator of anthocyanin biosynthesis in *Arabidopsis*, where its overexpression activated *DFR* and *ANS* gene expression, leading to anthocyanin accumulation [18]. Similar mechanisms have been reported in *Brassica napus* [40], *Zea mays* [41], and *Hordeum vulgare* [42]. Here, three AtMYB75 homologs—*NaMYB75a* (*Unigene0114694*), *NaMYB75b* (*Unigene0114295*), and *NaMYB75c* (*Unigene0107403*)—were identified in *N. atrans*, suggesting their potential regulatory involvement in flower color formation.

In addition to genetic and transcriptional regulation, plant hormones, especially the JA signaling pathway, play a key role in regulating anthocyanin biosynthesis. External application of methyl jasmonate (MeJA) reportedly elevates both total and specific anthocyanin levels in *Vitis vinifera* [24]. In our study, several key genes in the JA biosynthesis pathway, including 13-LOX, AOS, AOC, and OPR3, were significantly upregulated at D3 and D5, suggesting a synchronized activation of the JA biosynthesis pathway during flower color transition. Elevated JA levels may release MYC2 from repression by JAZ proteins. Notably, *MYC2* expression was markedly increased at D3 and D5, indicating activation of the JA signaling pathway. This study provides transcriptomic and metabolomic insights into anthocyanin accumulation during flower development in *N. atrans*. Our analyses suggest potential regulatory mechanisms involving MYB transcription factors and JA signaling pathway in the flavonoid pathway. Future studies using exogenous methyl jasmonate application or JA biosynthesis inhibitors are needed to establish causal relationships. In *N. atrans*, multiple candidate transcription factors were identified, comprising 12 MYB, 8 bHLH, and 6 WD40 proteins, all of which exhibit co-expression with anthocyanin biosynthetic genes throughout the flower color transition. Moreover, the coordinated upregulation of JA signaling genes and MBW complex components implies a potential interplay between the JA pathway and established anthocyanin regulators, possibly via MYC2-mediated activation of the MBW complex.

## 4. Materials and Methods

### 4.1. Plant Materials and Phenotypic Observation

The water lilies *N. atrans* used in this experiment were cultivated at the Haikou germplasm nursery of Hainan Fodu Lianyuan Ecological Agriculture Co., Ltd. Haikou has an annual mean temperature of approximately 24.2 °C, with extremes ranging from a maximum of 39.6 °C to a minimum of 2.8 °C, and receives more than 2000 h of sunshine annually. Plants were grown outdoors in ordinary pond mud under natural conditions, with a planting density of 2 m × 3 m and a water depth of 0.5–0.6 m. Samples were collected on 26 August 2020, at the full-bloom stage. Petal tissues were dissected from the upper-middle portion of the median whorl, rapidly frozen in liquid nitrogen, and maintained under cryogenic conditions until further processing. During anthesis, images of the flowers were captured using a digital camera, and color parameters (L*, a*, b*) in the CIELAB color space were measured using a colorimeter (Spectro P300, Datacolor Inc. Lawrenceville, New Jersey, USA). Observations and measurements were conducted continuously for five days. Petal samples were collected on day 1 (D1), day 3 (D3), and day 5 (D5) of flowering, representing the early, middle, and late stages of color transition, respectively. All specimens were promptly cryopreserved in liquid nitrogen and maintained at −80 °C pending downstream processing.

### 4.2. Targeted Metabolomics Analysis

Petal tissues from D1, D3, and D5 stages were subjected to metabolomic profiling, with three biological replicates for each time point. Metabolomic analyses were performed by Gene Denovo Biotechnology Co. (Guangzhou, China). Petal samples were freeze-dried and ground into fine powder, and 100 mg of powder was extracted with 70% methanol at 4 °C overnight. After centrifugation (10,000× *g*, 10 min), the supernatant was filtered through a 0.22 μm membrane for metabolomic analysis. A UPLC-ESI-MS/MS system (Shimadzu UFLC CBM30A with AB Sciex QTRAP 6500, Kobe, Japan) and a C18 column (2.1 mm × 100 mm, 1.8 μm) were used for metabolite analysis, with gradient elution at 0.4 mL/min and 40 °C [43]. Positive ion mode mass spectrometry, coupled with multiple reaction monitoring (MRM), was employed for metabolite detection and quantification. Quality control (QC) samples were included to ensure data reproducibility. Analyst 1.6.1 software was employed for peak detection, alignment, and quantification of raw data. Metabolite identification was achieved by comparing *m*/*z* values, retention times, and fragmentation profiles against in-house spectral libraries and public repositories (MassBank, HMDB, and METLIN) [44,45]. Differentially accumulated metabolites (DAMs) were defined by thresholds of VIP > 1.0, |fold change| > 2.0, and *p* < 0.05. KEGG pathway enrichment was subsequently performed using OmicShare tools [46]. Data represent mean ± SD (n = 3).

### 4.3. Transcriptome Analysis

Petal tissues from D1, D3, and D5 stages were used for transcriptome sequencing, with three biological replicates per stage, resulting in nine cDNA libraries. Sequencing was executed on an Illumina NovaSeq 6000 platform (Gene Denovo Biotechnology Co., Guangzhou, China). The raw sequence data are publicly accessible via the NCBI database under accession codes SRR34383805–SRR34383813. Raw reads were processed using the fastp software (v 0.20.0) to generate clean reads [47] and subsequently assembled de novo using the Trinity software (v1.1.3) [48]. Gene expression levels were quantified using featureCounts-8 [49], and transcript abundance was expressed as fragments per kilobase of transcript per million mapped reads (FPKM). Identification of DEGs was performed with DESeq2 software (R4.1.2) under the following parameters: |log_2_FC| ≥ 1 and FDR < 0.05 [50]. Data represent mean ± SD (n = 3). GO and KEGG enrichment analyses of DEGs were conducted using OmicShare tools [46].

### 4.4. RNA Isolation and RT-qPCR Analysis

Total RNA was isolated from petal samples with the DP432 RNAprep pure Plant Kit (Tiangen, Beijing, China), and for RT-qPCR, reverse transcription of total RNA was performed using the rr037 primescript™ rt reagent kit (Takara, Kusatsu, Shiga, Japan). Standard methodologies were adopted for RNA purification, reverse transcription, and RT-qPCR, wherein the Actin transcript served as an internal normalization standard [6]. Each experiment included three independent biological replicates, and each biological sample was analyzed in triplicate for technical replication. Relative transcript abundances were determined according to the 2^−ΔΔCt^ method [51]. Details of the RT-qPCR primers are summarized in Table S1.

### 4.5. Bioinformatics Analysis

Phylogenetic analyses and sequence alignments utilized MEGA-X (12.1) [52]. Phylogenetic tree is visualized by Evolview v3 website [53].

### 4.6. Statistical Analysis

R software (4.5.3) was employed to perform principal component analysis (PCA), orthogonal partial least squares discriminant analysis (OPLS-DA), clustering, and correlation analyses. Violin plots and bubble charts were generated using R software, while Venn plots, upset plots, and heatmaps were constructed with TBtools-II software (1.04) [54].

## 5. Conclusions

The progressive color change from near-white to rose-red observed in *N. atrans* coincided with substantial buildup of seven anthocyanin species and transcriptional induction of numerous essential enzyme-encoding genes involved in the anthocyanin biosynthetic route. The activation of the anthocyanin biosynthetic pathway and sustained anthocyanin accumulation were identified as the direct drivers of the flower color transition. Simultaneously, genes implicated in JA biosynthesis along with core regulators of the JA signaling cascade were substantially induced, implying a potential involvement of JA signaling in the control of anthocyanin production. The present work advances our understanding of the metabolic and molecular mechanisms underpinning flower color change in *N. atrans*, thereby providing a theoretical framework for genetic improvement of floral coloration in water lily cultivars.

## Data Availability

All data supporting this study are available within the paper and within the Supplementary Materials published online.

## Supplementary Materials

The following supporting information can be downloaded at: https://www.mdpi.com/article/10.3390/genes17040442/s1, Figure S1: Multivariate statistical analysis of metabolomics; Table S1: Primers sequence for RT-qPCR analysis.

## References

[1] Fan Y., Jin X., Wang M., Liu H., Tian W., Xue Y., Wang K., Li H., Wu Y. (2024). Flower morphology, flower color, flowering and floral fragrance in *Paeonia* L.. Front. Plant Sci..

[2] Zhao D., Tao J. (2015). Recent advances on the development and regulation of flower color in ornamental plants. Front. Plant Sci..

[3] Ai Y., Zheng Q.D., Wang M.J., Xiong L.W., Li P., Guo L.T., Wang M.Y., Peng D.H., Lan S.R., Liu Z.J. (2023). Molecular mechanism of different flower color formation of *Cymbidium ensifolium*. Plant Mol. Biol..

[4] Xie C., Tian Q., Qiu H., Wang R., Wang L., Yue Y., Yang X. (2024). Methylation modification in ornamental plants: Impact on floral aroma and color. Int. J. Mol. Sci..

[5] Zhang L., Chen F., Zhang X., Li Z., Zhao Y., Lohaus R., Chang X., Dong W., Ho S.Y.W., Liu X. (2020). The water lily genome and the early evolution of flowering plants. Nature.

[6] Liu Q., Li S., Li T., Wei Q., Zhang Y. (2024). The characterization of R2R3-MYB genes in water lily *Nymphaea colorata* reveals the involvement of NcMYB25 in regulating anthocyanin synthesis. Plants.

[7] Hu X., Liang Z., Sun T., Huang L., Wang Y., Chan Z., Xiang L. (2024). The R2R3-MYB transcriptional repressor TgMYB4 negatively regulates anthocyanin biosynthesis in tulips (*Tulipa gesneriana* L.). Int. J. Mol. Sci..

[8] Alappat B., Alappat J. (2020). Anthocyanin pigments: Beyond aesthetics. Molecules.

[9] Zhao Y., Jiang C., Lu J., Sun Y., Cui Y. (2023). Research progress of proanthocyanidins and anthocyanidins. Phytother. Res..

[10] Tanaka Y., Brugliera F., Kalc G., Senior M., Dyson B., Nakamura N., Katsumoto Y., Chandler S. (2010). Flower color modification by engineering of the flavonoid biosynthetic pathway: Practical perspectives. Biosci. Biotechnol. Biochem..

[11] Liu W., Feng Y., Yu S., Fan Z., Li X., Li J., Yin H. (2021). The flavonoid biosynthesis network in plants. Int. J. Mol. Sci..

[12] Yan H., Pei X., Zhang H., Li X., Zhang X., Zhao M., Chiang V.L., Sederoff R.R., Zhao X. (2021). MYB-mediated regulation of anthocyanin biosynthesis. Int. J. Mol. Sci..

[13] Ramsay N.A., Glover B.J. (2005). MYB-bHLH-WD40 protein complex and the evolution of cellular diversity. Trends Plant Sci..

[14] Zhao L., Gao L., Wang H., Chen X., Wang Y., Yang H., Wei C., Wan X., Xia T.J.F. (2013). The R2R3-MYB, bHLH, WD40, and related transcription factors in flavonoid biosynthesis. Funct. Integr. Genom..

[15] Liu Q., Zhao Y., Wang Y., Wei Q., Zhang Y., Li S. (2025). Comprehensive genome-wide analysis of bHLH family genes in *Nymphaea colorata* and molecular characterization of NcTT8. BMC Plant Biol..

[16] Liu Y., Ma K., Qi Y., Lv G., Ren X., Liu Z., Ma F. (2021). Transcriptional regulation of anthocyanin synthesis by MYB-bHLH-WDR complexes in kiwifruit (*Actinidia chinensis*). J. Agric. Food Chem..

[17] Qi T., Song S., Ren Q., Wu D., Huang H., Chen Y., Fan M., Peng W., Ren C., Xie D. (2011). The Jasmonate-ZIM-domain proteins interact with the WD-Repeat/bHLH/MYB complexes to regulate Jasmonate-mediated anthocyanin accumulation and trichome initiation in *Arabidopsis thaliana*. Plant Cell.

[18] Zuluaga D.L., Gonzali S., Loreti E., Pucciariello C., Degl’Innocenti E., Guidi L., Alpi A., Perata P. (2008). *Arabidopsis thaliana* MYB75/PAP1 transcription factor induces anthocyanin production in transgenic tomato plants. Funct. Plant Biol..

[19] Kreynes A.E., Yong Z., Liu X.-M., Wong D.C.J., Castellarin S.D., Ellis B.E. (2020). Biological impacts of phosphomimic AtMYB75. Planta.

[20] Li Z., Ahammed G.J. (2023). Hormonal regulation of anthocyanin biosynthesis for improved stress tolerance in plants. Plant Physiol. Biochem..

[21] Li T., Jia K.-P., Lian H.-L., Yang X., Li L., Yang H.-Q. (2014). Jasmonic acid enhancement of anthocyanin accumulation is dependent on phytochrome a signaling pathway under far-red light in *Arabidopsis*. Biochem. Biophys. Res. Commun..

[22] Zhang Z., Chen C., Jiang C., Lin H., Zhao Y., Guo Y. (2024). VvWRKY5 positively regulates wounding-induced anthocyanin accumulation in grape by interplaying with VvMYBA1 and promoting jasmonic acid biosynthesis. Hortic. Res..

[23] Wang S., Li L.-X., Fang Y., Li D., Mao Z., Zhu Z., Chen X.-S., Feng S.-Q. (2022). MdERF1B–MdMYC2 module integrates ethylene and jasmonic acid to regulate the biosynthesis of anthocyanin in apple. Hortic. Res..

[24] Ju Y.-L., Liu M., Zhao H., Meng J.-F., Fang Y.-L. (2016). Effect of exogenous abscisic acid and methyl jasmonate on anthocyanin composition, fatty acids, and volatile compounds of cabernet sauvignon (*Vitis vinifera* L.) grape berries. Molecules.

[25] An J.P., Xu R.R., Wang X.N., Zhang X.W., You C.X., Han Y. (2024). MdbHLH162 connects the gibberellin and jasmonic acid signals to regulate anthocyanin biosynthesis in apple. J. Integr. Plant Biol..

[26] Yoshida K., Miki N., Momonoi K., Kawachi M., Katou K., Okazaki Y., Uozumi N., Maeshima M., Kondo T. (2009). Synchrony between flower opening and petal-color change from red to blue in morning glory, *Ipomoea tricolor* cv. Heavenly Blue. Proc. Jpn. Acad. B.

[27] Yoshida K., Kawachi M., Mori M., Maeshima M., Kondo M., Nishimura M., Kondo T. (2005). The involvement of tonoplast proton pumps and Na+(K+)/H+ exchangers in the change of petal color during flower opening of morning glory, *Ipomoea tricolor* cv. Heavenly blue. Plant Cell Physiol..

[28] Zhu Z., Zeng X., Shi X., Ma J., Liu X., Li Q. (2023). Transcription and metabolic profiling analysis of three discolorations in a day of *Hibiscus mutabilis*. Biology.

[29] Yang Y., Liu X., Shi X., Ma J., Zeng X., Zhu Z., Li F., Zhou M., Guo X., Liu X. (2022). A high-quality, chromosome-level genome provides insights into determinate flowering time and color of cotton rose (*Hibiscus mutabilis*). Front. Plant Sci..

[30] Wu Q., Li P.C., Zhang H.J., Feng C.Y., Li S.S., Yin D.D., Tian J., Xu W.Z., Wang L.S. (2018). Relationship between the flavonoid composition and flower colour variation in *Victoria*. Plant Biol..

[31] Wen X., Liang Y., Shan H., Chang X., Song X., Shen S., Fu Y., Chen D., Chen F., Li Y. (2025). The genome of giant waterlily provides insights into the origin of angiosperms, leaf gigantism, and stamen function innovation. Plant Commun..

[32] Yang G., Wei J., Wu Y., Chen S., Yu C., Zhu Y., Lin Z., Lv H., Chen Y. (2024). Comprehensive study of non-volatile and volatile metabolites in five water lily species and varieties (*Nymphaea* spp.) using widely targeted metabolomics. Beverage Plant Res..

[33] Zhou X., Wang X., Wei H., Zhang H., Wu Q., Wang L. (2024). Integrative analysis of transcriptome and target metabolites uncovering flavonoid biosynthesis regulation of changing petal colors in *Nymphaea* ‘Feitian 2’. BMC Plant Biol..

[34] Parrish S.B., Paudel D., Deng Z. (2023). Transcriptome analysis of *Lantana camara* flower petals reveals candidate anthocyanin biosynthesis genes mediating red flower color development. G3 Genes Genomes Genet..

[35] Ye L.J., Möller M., Luo Y.H., Zou J.Y., Zheng W., Wang Y.H., Liu J., Zhu A.D., Hu J.Y., Li D.Z. (2021). Differential expressions of anthocyanin synthesis genes underlie flower color divergence in a sympatric *Rhododendron sanguineum* complex. BMC Plant Biol..

[36] Liu X.F., Teng R., Xiang L., Li F., Chen K. (2023). Sucrose-delaying flower color fading associated with delaying anthocyanin accumulation decrease in cut chrysanthemum. PeerJ.

[37] Zhao D., Jiang Y., Ning C., Meng J., Lin S., Ding W., Tao J. (2014). Transcriptome sequencing of a chimaera reveals coordinated expression of anthocyanin biosynthetic genes mediating yellow formation in herbaceous peony (*Paeonia lactiflora* Pall.). BMC Genom..

[38] Schwinn K.E., Ngo H., Kenel F., Brummell D.A., Albert N.W., McCallum J.A., Pither-Joyce M., Crowhurst R.N., Eady C., Davies K.M. (2016). The onion (*Allium cepa* L.) R2R3-MYB gene myb1 regulates anthocyanin biosynthesis. Front. Plant Sci..

[39] Li W., Ding Z., Ruan M., Yu X., Peng M., Liu Y. (2017). Kiwifruit R2R3-MYB transcription factors and contribution of the novel AcMYB75 to red kiwifruit anthocyanin biosynthesis. Sci. Rep..

[40] Xie T., Zan X., Chen X., Zhu H., Rong H., Wang Y., Jiang J. (2022). An R3-MYB repressor, BnCPC forms a feedback regulation with MBW complex to modulate anthocyanin biosynthesis in *Brassica napus*. Biotechnol. Biofuels Bioprod..

[41] Qin L., Sun L., Wei L., Yuan J., Kong F., Zhang Y., Miao X., Xia G., Liu S. (2021). Maize SRO1e represses anthocyanin synthesis through regulating the MBW complex in response to abiotic stress. Plant J..

[42] Chen L., Cui Y., Yao Y., An L., Bai Y., Li X., Yao X., Wu K. (2023). Genome-wide identification of WD40 transcription factors and their regulation of the MYB-bHLH-WD40 (MBW) complex related to anthocyanin synthesis in Qingke (*Hordeum vulgare* L. var. nudum Hook. f.). BMC Genom..

[43] Chen W., Gong L., Guo Z., Wang W., Zhang H., Liu X., Yu S., Xiong L., Luo J. (2013). A novel integrated method for large-scale detection, identification, and quantification of widely targeted metabolites: Application in the study of rice metabolomics. Mol. Plant.

[44] Wishart D.S., Jewison T., Guo A.C., Wilson M., Knox C., Liu Y., Djoumbou Y., Mandal R., Aziat F., Dong E. (2013). HMDB 3.0--The Human Metabolome Database in 2013. Nucleic Acids Res..

[45] Zhu Z.-J., Schultz A.W., Wang J., Johnson C.H., Yannone S.M., Patti G.J., Siuzdak G. (2013). Liquid chromatography quadrupole time-of-flight mass spectrometry characterization of metabolites guided by the METLIN database. Nat. Protoc..

[46] Mu H., Chen J., Huang W., Huang G., Deng M., Hong S., Ai P., Gao C., Zhou H. (2024). OmicShare tools: A zero-code interactive online platform for biological data analysis and visualization. iMeta.

[47] Chen S., Zhou Y., Chen Y., Gu J. (2018). fastp: An ultra-fast all-in-one FASTQ preprocessor. Bioinformatics.

[48] Haas B.J., Papanicolaou A., Yassour M., Grabherr M., Blood P.D., Bowden J., Couger M.B., Eccles D., Li B., Lieber M. (2013). De novo transcript sequence reconstruction from RNA-seq using the Trinity platform for reference generation and analysis. Nat. Protoc..

[49] Liao Y., Smyth G.K., Shi W. (2014). featureCounts: An efficient general purpose program for assigning sequence reads to genomic features. Bioinformatics.

[50] Love M.I., Huber W., Anders S. (2014). Moderated estimation of fold change and dispersion for RNA-seq data with DESeq2. Genome Biol..

[51] Livak K.J., Schmittgen T.D. (2001). Analysis of relative gene expression data using real-time quantitative PCR and the 2^−ΔΔCT^ method. Methods.

[52] Kumar S., Stecher G., Li M., Knyaz C., Tamura K. (2018). MEGA X: Molecular evolutionary genetics analysis across computing platforms. Mol. Biol. Evol..

[53] Subramanian B., Gao S., Lercher M.J., Hu S., Chen W.H. (2019). Evolview v3: A webserver for visualization, annotation, and management of phylogenetic trees. Nucleic Acids Res..

[54] Chen C., Wu Y., Li J., Wang X., Zeng Z., Xu J., Liu Y., Feng J., Chen H., He Y. (2023). TBtools-II: A “one for all, all for one” bioinformatics platform for biological big-data mining. Mol. Plant.

