# Supplementary material for: Unraveling the Metabolic and Molecular Basis of Floral Pigmentation Shift in Nymphaea atrans
Source: Genes (Basel). 2026 Apr 12;17(4):442. doi: 10.3390/genes17040442 (PMC13115826; doi:10.3390/genes17040442)
Supplement: Supplementary file 1 [file genes-17-00442-s001.zip › FIgure S1.pdf]

Supplementary Figures

Unraveling the Metabolic and Molecular Basis of Floral Pigmentation Shift in *Nymphaea atrans*

Qian Wei [1], Kaijie Zhou [2], Mengchao Fang [3], Zhentao Ren [4], Shujuan Li [1], Ming Zhu [5,*]

1 Xi'an Botanical Garden of Shaanxi Province, Institute of Botany of Shaanxi Province, Shaanxi Engineering Research Centre for Conservation and Utilization of Botanical Resources, Xi'an, 710061, China.

2 College Biological Science and Food Engineering, Southwest Forestry University, Kunming, 650224, China.

3 School of Geography and Planning, Chizhou University, Chizhou, 247000, China.

4 Institute of Food and Nutrition Development, Ministry of Agriculture and Rural Affairs , Beijing 100081, China.

5 Yunnan Open University, Kunming, 650000, China; lyanzhu0@126.com.

* Correspondence: lyanzhu0@126.com; Tel.: +86-0871-65100038

Supplementary Figures Description

This document contains supplementary figures related to the manuscript titled " Metabolic and Molecular Mechanisms Underlying Flower Color Transition in *Nymphaea atrans*". These figures are intended to provide additional visual support and clarification for the results presented in the study. Each figure is clearly labeled and includes a brief description to aid in understanding the research findings.

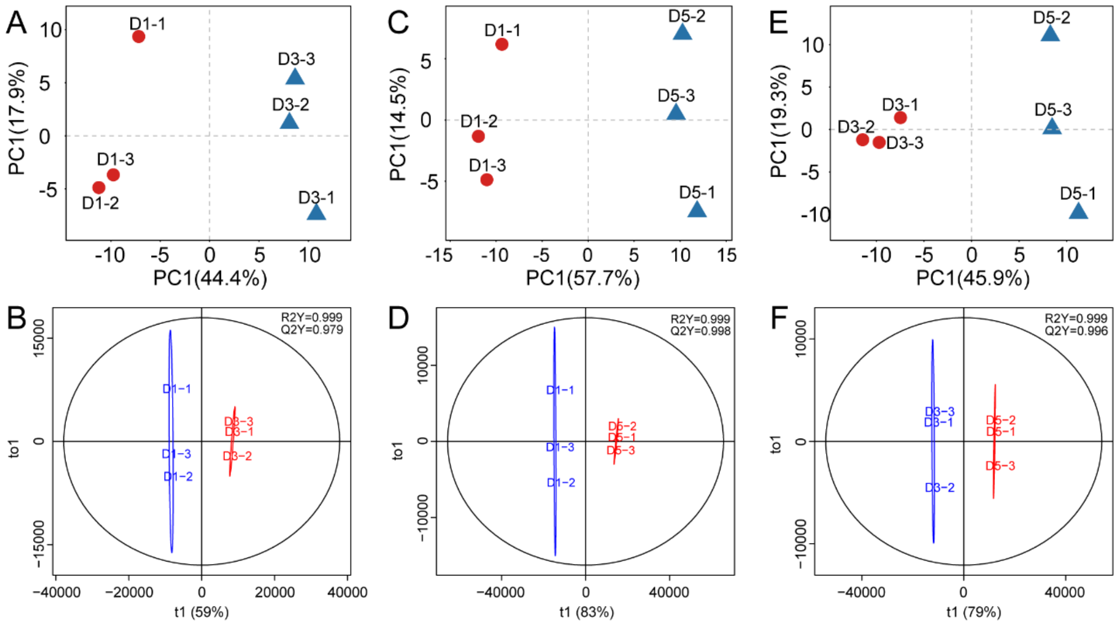

Figure S1. Multivariate statistical analysis of metabolomics
