# Supplementary material for: Unraveling the Metabolic and Molecular Basis of Floral Pigmentation Shift in Nymphaea atrans
Source: Genes (Basel). 2026 Apr 12;17(4):442. doi: 10.3390/genes17040442 (PMC13115826; doi:10.3390/genes17040442)
Supplement: Supplementary file 1 [file genes-17-00442-s001.zip › Table S1.pdf]

Table S1 Primers sequence for RT-qPCR analysis

Gene	Primer sequence (5'→3')
Unigene0004067-AS	CGCAAGCCCGAAGCCGAATT
Unigene0004067-S	ACCAACCTGCACGCCAACATC
Unigene0008478-AS	GCGGGCACTTGGGATAGTAGTT
Unigene0008478-S	CTGAGGAAGGTGGTGACGAAGA
Unigene0008908-AS	GCTTCTCAGGCTTCAAGGACCA
Unigene0008908-S	CCAACTCCGTCGTCTACCTCAG
Unigene0009151-AS	GCTCGCATCTTCTCGGTCTTGA
Unigene0009151-S	TCGGCATCTCGGACTGGAACT
Unigene0014307-AS	GGCGTCGTTGTGCTTCAGGAA
Unigene0014307-S	CACGATGCCTCACCACTCACTC
Unigene0069057-AS	ATCGCCTTCCGCCTTGTATTCC
Unigene0069057-S	GTGCAGTGCTGCCAGGAGAA
Unigene0078775-AS	ACGGCGAAGTTGATGGCAGAAG
Unigene0078775-S	AAGGACCTCGACGGCAAGAAGT
Unigene0091380-AS	GCAGTGTTGGATGAGGAAGGGA
Unigene0091380-S	TCAATTGGAGAGCGCAGTGAGG
Unigene0095465-AS	TGTCGAGTTCCAGCCGCAGT
Unigene0095465-S	TCAAGTCGGAGGAGTCGGTTCG
Unigene0116834-AS	CGTTGCTGAAGTCGTTGTAGGC
Unigene0116834-S	CAACAGTGGTGGTGGAGTCGTC
Unigene0122715-AS	GCCTGTTGGTGAAGGTGGTGTC
Unigene0122715-S	CCGTCATAGGTTGCCTGCCATC
